# MXene nanomaterials in biomedicine: A bibliometric perspective

**DOI:** 10.3389/fbioe.2023.1184275

**Published:** 2023-04-19

**Authors:** Runying Guo, Daorun Hu, Danrui Liu, Qingkun Jiang, Jiaxuan Qiu

**Affiliations:** ^1^ Department of Stomatology, First Affiliated Hospital of Nanchang University, Nanchang, China; ^2^ Medical College, Nanchang University, Nanchang, China

**Keywords:** MXene, biomaterials, biological application, bibliometric review, visualization

## Abstract

**Purpose:** MXene is two-dimensional (2D) nanomaterials that comprise transition metal carbides, nitrides, and carbonitrides. Their unique nanostructure attributes it a special role in medical applications. However, bibliometric studies have not been conducted in this field. Therefore, the aim of the present study was to conduct a bibliometric analysis to evaluate the global scientific output of MXene in biomedical research, explore the current situation of this field in the past years and predicte its research hotpots.

**Methods:** We utilized visual analysis softwares Citespace and Bibliometrix to analyze all relevant documents published in the period of 2011–2022. The bibliometric records were obtained from the Web of Science Core Collection.

**Results:** A total of 1,489 publications were analyzed in this study. We observed that China is the country with the largest number of publications, with Sichuan University being the institution with the highest number of publications in this field. The most publications on MXene medicine research in the past year were found primarily in journals about Chemistry/Materials/Physics. Moreover, ACS Applied Materials and Interfaces was found to be the most productive journal in this field. Co-cited references and keyword cluster analysis revealed that #antibacterial# and #photothermal therapy# are the research focus keyword and burst detection suggested that driven wearable electronics were newly-emergent research hot spots.

**Conclusion:** Our bibliometric analysis indicates that research on MXene medical application remains an active field of study. At present, the research focus is on the application of MXene in the field of antibacterial taking advantage of its photothermal properties. In the future, wearable electronics is the research direction of MXene medical application.

## 1 Introduction

MXene is a novel two-dimensional (2D) nanomaterial composed of transition metal carbides and nitrides or carbonitrides. Since its initial discovery by Gogotsi in 2011, MXene has been widely used in various fields, including energy storage, catalysis, and conversion (Zhong et al., 2022). The application of MXene in supercapacitors enhance its volume specific capacitance (Liu et al., 2023). When used in lithium-sulfur batteries, MXene can improve their cycling performance. (Wang et al., 2023). MXene can be used in photocatalytic field by schottky junctions and heterojunctions (Xi et al., 2023).

As a new type of 2D nanomaterial, MXene inherits many advantages of common 2D materials, including large specific surface area and excellent electronic, mechanical, and physicochemical properties (Huang et al., 2020). Besides these properties, researchers have found additional characteristics of MXene that make them appropriate for biomedical applications: 1) at the surface of MXene, there are many functional groups, such as hydroxyl, oxygen or fluorine; this allows researchers to load different substances on the surface of MXene, such as various drugs and hydrophilic macromolecules (Guo et al., 2022; Ye et al., 2022; Yu et al., 2022); 2) unlike hydrophobic nanomaterials such as graphene, MXene is hydrophilic, which makes it biocompatible (Klimkevicius et al., 2022; Perumal et al., 2022); 3) MXene exhibits the characteristics of near-infrared absorption (NIR), which enables its application in photothermal therapy (PTT) and photoacoustic imaging (PTA) fields (Huang et al., 2020). Based on the abovementioned excellent properties of MXene, researchers have used it in various fields such as microbiology, oncology, and tissue engineering (Du et al., 2022; Murali et al., 2021; Zhang Z. et al., 2022b). For example, Min Zhang and others have previously reported the use of 2D nanosonosensitizers/nanocatalysts (Ti_3_C_2_/CuO_2_@BSA) for efficient and synergistic acoustic/chemo-dynamic tumor therapy through the generation of nanosensitizers *in situ* (Zhang M. et al., 2022a). Li et al. designed a nanosensitizer based on hyaluronic acid grafted dopamine (HA-DA) and polydopamine (PDA)-coated Ti_3_C_2_ MXene nanosheets in an injectable hydrogel. Indeed, MXene anchored hydrogels not only exert antioxidant and antibacterial effects but also have multifunctional properties such as tissue adhesion, self-healing, injectability and hemostasis, which significantly enhance diabetic wound healing (Li Y. et al., 2022c).

The biological applications of MXene have been discussed in numerous review publications, however, these mainly focus on the current status of overall biological applications (Huang et al., 2018; Koyappayil et al., 2022; Parajuli et al., 2022) or only address a specific biomedical application such as their use as antibacterial agents, biosensors or for cancer imaging and treatment (Ali et al., 2022; Hao et al., 2022; Ranjbari et al., 2022). Bibliometric analysis is a research method that statistically evaluates and visually presents the current state-of-the-art, most influential studies and upcoming trends of a given field of research (Yang et al., 2022). Through quantitative and qualitative analysis of different metrics such as countries, institutional authors, keywords, etc. of data present in published journals, bibliometric analysis not only enables people to clearly visualize the changes of past research hotspots, the most influential countries/institutions and authors in a given field, but also to predict future hotspots and trends through the available information. Currently, many biomedical applications of nanomaterials have been well studied and explored through bibliometric analysis. For example, Gu et al. (2022) conducted a bibliometric analysis of tungsten-based nanomaterials (TNM) bioapplications to explore the prospects and challenges of TNM in biomedical research, discussing the development of TNM from another perspective. Similarly, Zhu et al. (2022) used VOSviewer to identify the biomedical fields related to 2D nanomaterials (e.g., “graphene oxide,” MoS_2_, “black phosphorus”), providing a holistic perspective about past decade developments. This kind of analytical method is becoming crucial for formulating policies and identifying current research trends. However, to date, no study has ever objectively summarized or analyzed research trends in MXene research and bioapplications, especially from a bibliometric perspective. Thus, this study uses the bibliometric method to conduct a macro review of the existing literature, study global research trends from 2011 (when MXene was first proposed (Huang et al., 2018)) to 2022, and predict possible future research hot spots (Table 1). This unique and objective perspective might be used as a relevant primer for future analysis in an exceedingly additional sensible direction.

**TABLE 1:** Employed search strategies.

Search set	Search term in WoS core collection
#1	TS = (MXene* or “transition metal carbide*” or ti3c2* or “2 dimensional titanium carbide” or “2D titanium carbide” or Ti2C* or Nb2C* or V2C* or Ti3CN* or Ti2N* or Nb4C3* or Ta4C3* or Cr2C* or TiNbC* or Ta2C* or Ti4N3*)
#2	TS = (medic* or biomedic* or nanomedic* or cancer or tumor or disease or biosens* or immunosens* or diagnos* or bioimaging or *therap* or theranos* or “drug deliver*” or “tissue engineering” or antibacteri* or antimicrobe* or antifouling or bioelectronic* or “wearable device*” or “electronic skin” or “health monitor*” or “human breath monitoring” or healthcare or biodevice* or bioassay* or “medical device” or “stem cell engineering” or biomarker or “biological application” or “medical application” or “biomedical application”)
#3	Language: English
	Period: 2011-01-01/2022-12-31
	Article type: Articles
#4	#1 and #2 and #3

## 2 Methods

### 2.1 Collect relevant documents

Data were obtained from the Web of Science (WoS) core collection from a period comprised between 1 January 2011 to 31 December 2022. We obtained a total of 1,489 publications to accurately present the development trajectory and cutting-edge research of MXene bioapplications, with refined search methods (Table 1).

### 2.2 Bibliometric analyses

In this study, CiteSpace (version 6.1. R6) and the Bibliometrix based on the R language were used for bibliometric analysis.

CiteSpace is a JAVA-based software developed for scientometric knowledge mapping research (Zhong et al., 2021), formulated by Professor Chaomei Chen. Based on existing literature, it can guide the visualization of research hotspots and evolutionary processes of each field and predict the development trend of each field (Li et al., 2022b; Ma et al., 2021). Such an efficient tool used for the analyses of large-scale information, it is able to explore and visually present significant events and trends of a given field and has been wide employed in numerous analysis fields (Ma et al., 2022).

Bibliometrix is associate open supply analysis tool for scientific and bibliometric quantitative analysis developed by Massimo Aria and Corrado Cuccurullo (Oyewola and Dada, 2022). It has the unique advantage of being easily operatable, which not only avoids tedious multi-step operations on the software processing interface for the user but also increases efficiency and reduces error probability. It allows batch analysis and highly repetitive and multi-step computational tasks with relative ease.

We imported the data obtained from WoS core collection into CiteSpace and Biblimetrix for analysis. The general bibliometric distribution was first performed according to the dataset of year, country, research institution, author, references and keyword. Then, based on the determined keyword analysis, we further analyzed research trends and hotspots of MXene medical applications to highlight a putative direction for the long-term development and clinical translation of MXene technology.

## 3 Results

### 3.1 General description

**FIGURE 1:**
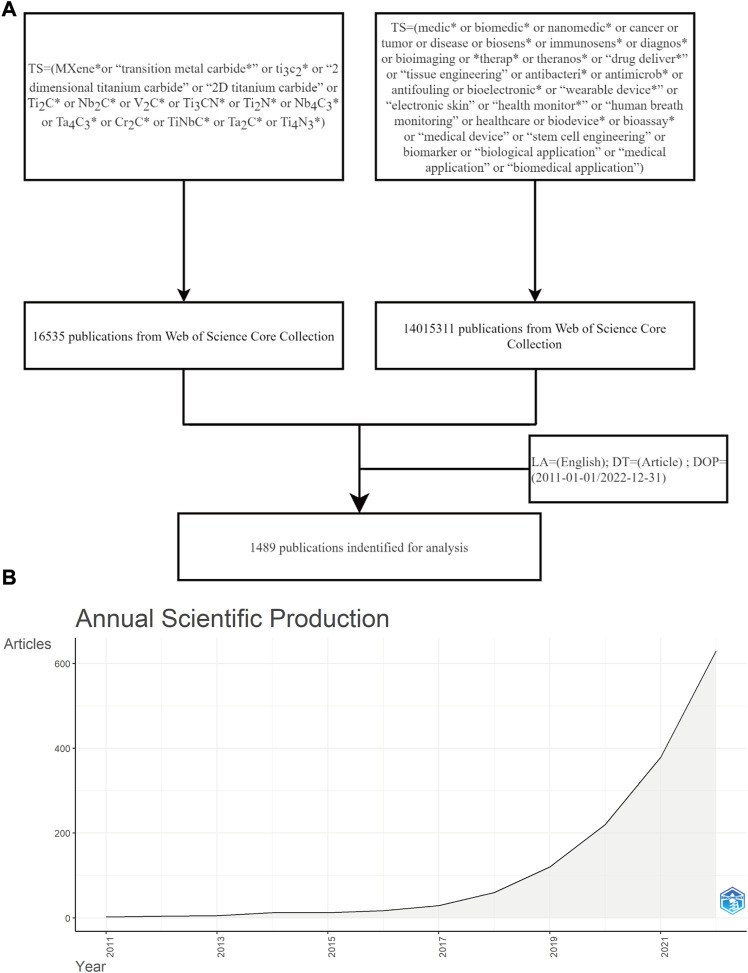
Data collection and overall distribution of publication output. **(A)** Literature screening flowchart. **(B)** Annual scientific production trends.

Based on the employed search strategies, we obtained a total of 1,489 articles from the WoS core collection (Figure 1A). As shown in Figure 1B, there has been an increase in the number of papers published on MXene bioapplications in the last years. Interestingly, the cumulative publication record from 2011 to 2018 was only 9.471% whereas the number of publications exceeded a hundred in 2019, accounting for 8.059% in that year alone (Supplementary Table S1). The number of publications in 2021 and 2022 was 379 and 629, respectively, accounting for 25.453% and 42.243% of the total number of publications, respectively. This increase in scientific production since 2019 is associated with the new innovations revolving around MXene synthesis (Saravanan et al., 2022). Overall, these results showed that the biological application of MXene has triggered extensive interest from medical researchers worldwide.

### 3.2 Country/region analysis

**FIGURE 2:**
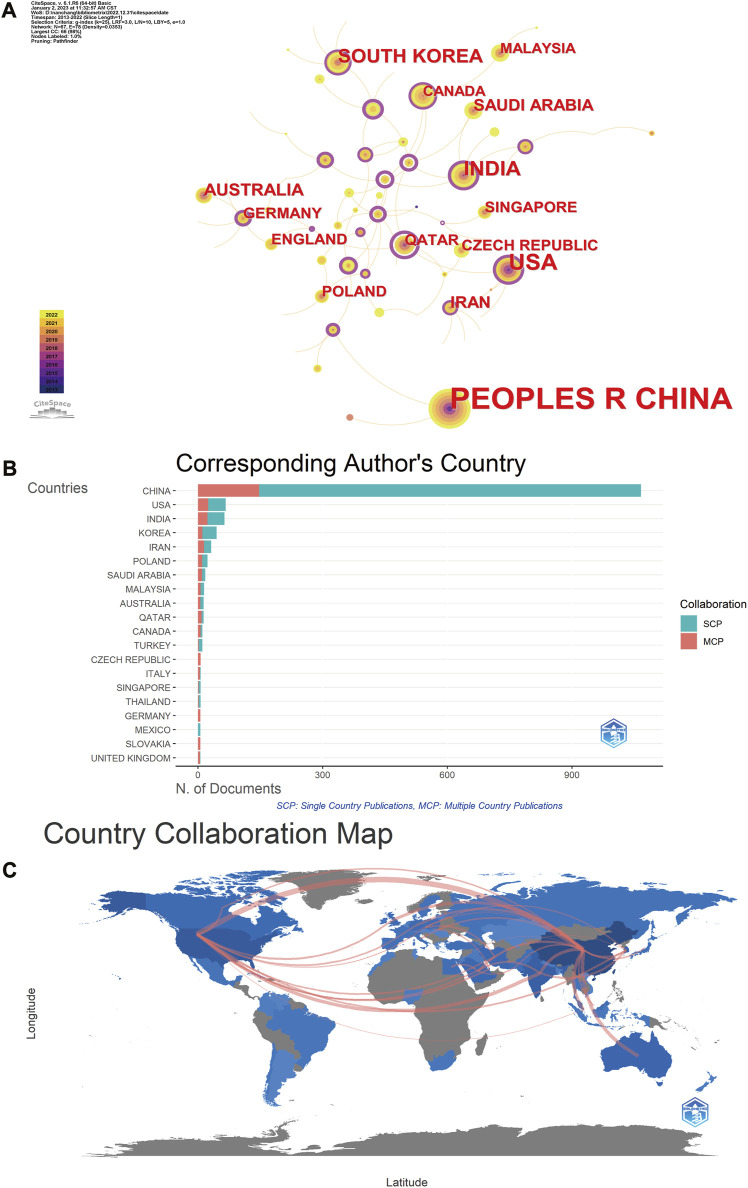
Visualization of the network map of countries. **(A)** Country distribution of MXene medical application. **(B)** Cooperation in the country where the corresponding author is located. **(C)** Map of cooperation between countries. SCP, single country publications; MCP, multiple country publications.

We used CiteSpace to examine the current state-of-art of articles on MXene medical applications by each country or region. The country/region distribution map consisted of sixty-seven nodes, which indicated that sixty-seven countries or regions have contributed to the analysis on biological applications of MXene (Figure 2A). The size of the node represents the number of documents issued by each country. The purple color in the outer circle of the node represents the high centrality of the country (centrality number >0.1), which means that the country has a large international influence. Results showed that PEOPLES R CHINA (China) ranks first (*n* = 1,079) by a substantial margin, followed by the United States (United States) (*n* = 160), INDIA (India) (*n* = 96). Although China has published a large number of papers on the medical application of MXene, its central figure is only 0.06, and its articles are mainly published in domestic cooperation (Figure 2B), which indicates that China has yet to invest on forming its own international cooperation network and strengthen academic exchanges with other countries. The United States, which ranks second in the number of publications, has a high centrality (0.34). This means that United States plays an important role in this research field and has formed its own international cooperation networks (Figure 2C).

### 3.3 Author and institution analysis

**FIGURE 3:**
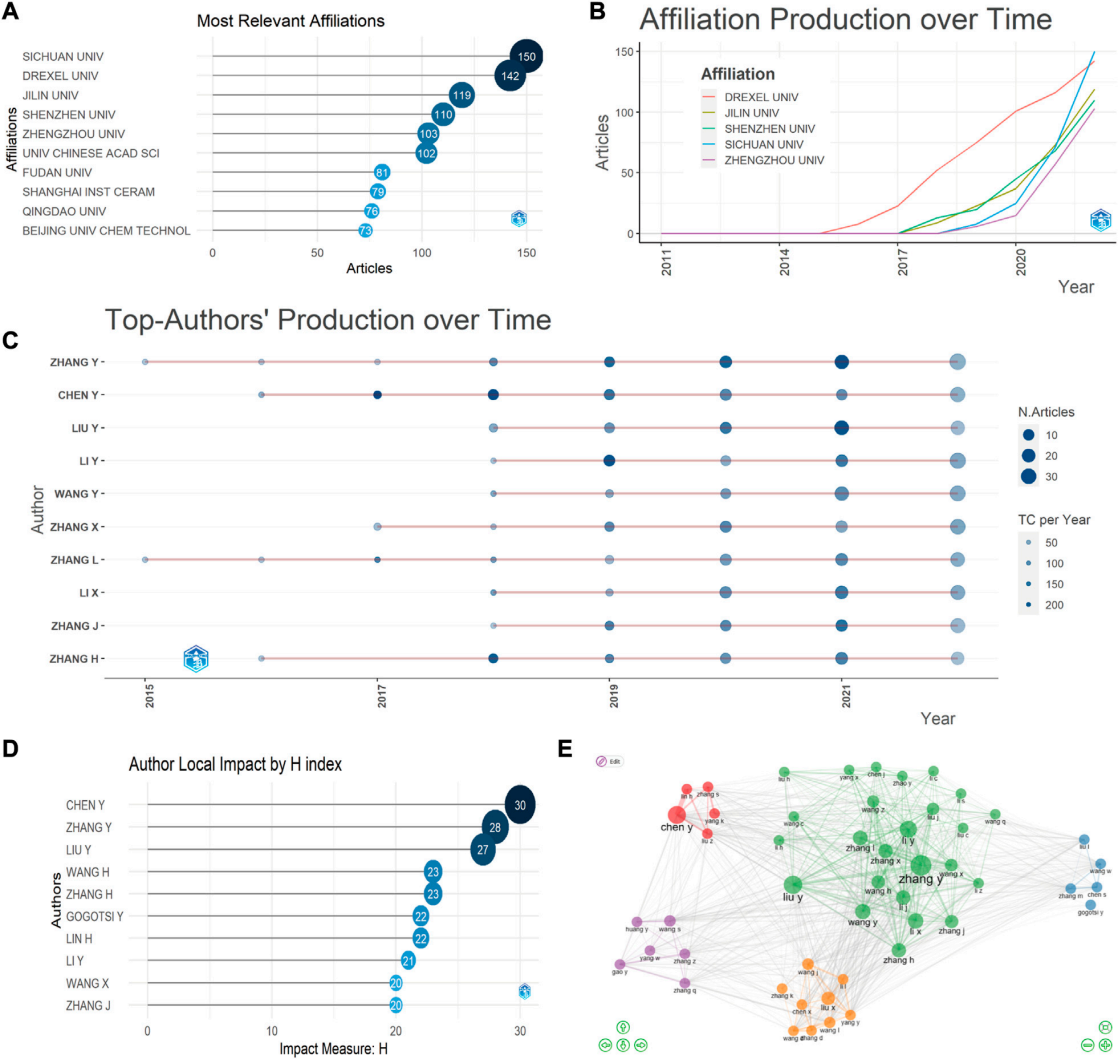
Image network of institutes and author. **(A)** Documents issued by active institutions, **(B)** Number of documents issued by active institutions over time, **(C)** Over time, the production of top authors dedicated to MXene medical applicatio. **(D)** H index sorting results of top writers. **(E)** Visualization network map of cooperation among authors.

We performed a co-occurrence analysis of research institutions using CiteSpace and obtained a total of 327 nodes, which implied that 327 research units actively contributed to investigation in MXene bioapplications (Supplementary Figure S1). The top 10 organizations with the most documents are shown in Figure 3A. In 2022, the number of documents issued by Sichuan University exceeded that of Drexel University for the first time, and the cumulative number of documents issued reached 150 (Figure 3B). In addition, nine of the ten institutions with the most publications were from China. This result also indicates that China plays a significant role in advancing and developing the field.

Many scholars are dedicated to explorative work and investigative study of MXene in the biomedical field. Author analysis in Figure 3C indicated that Zhang Y has published most papers in this regard, followed by Chen Y and Liu Y. H-index analysis in Figure 3D indicated that the h-index of publications is highest for Chen Y (30), followed by Zhang Y (28), Liu Y (27), Wang H (23), Zhang H (23), Gogotsi Y (22), Lin H (22), Li Y (21), Wang X (20), and Zhang J (20) (Figure 3D). Overall, the most influential authors in biomedical applications of MXene were from China. These top ten authors belonged to different research areas, from biosensors and antibacterial activity to bioimaging, therapeutics, and theranostics.

Author collaboration network analysis is a method used to assess the interaction between researchers (Khalagi et al., 2021). We performed a collaborative building block map of authors by Bibliometrix and the results showed that Zhang Y, the most prolific author in this area of research, has made greater contribution to the advancement of the discipline through cooperation with several authors (Figure 3E).

Co-cited authors are those who have been co-cited in a series of publications (Ke et al., 2020). Analysis of co-citations by CiteSpace revealed that Lin H ranked first with 305 co-citations, followed by Anasori B (*n* = 259) and Alhabeb M (*n* = 236) (Supplementary Figure S2).

### 3.4 Journal analysis

**FIGURE 4:**
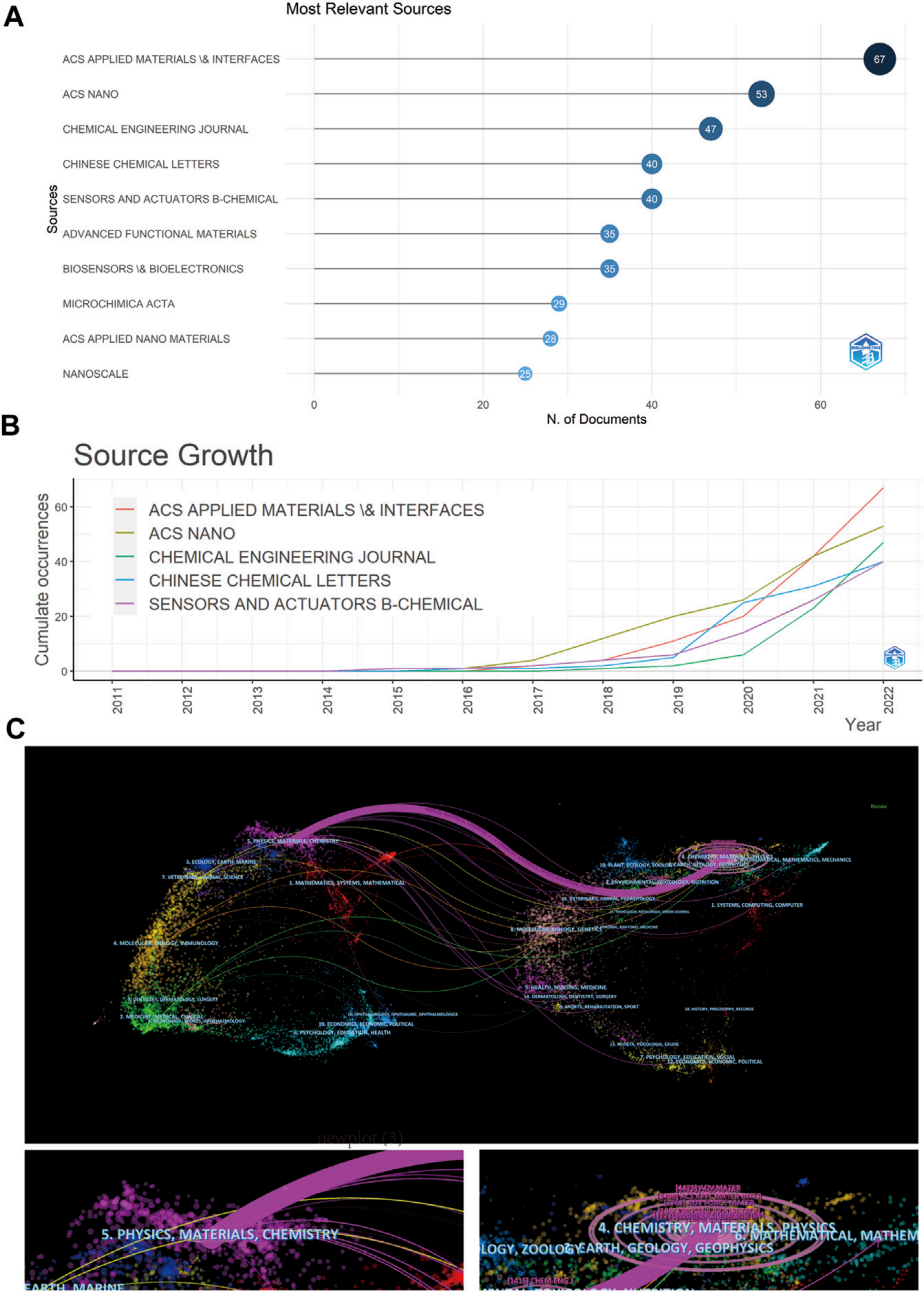
Published journals of MXene medical application **(A)** The journal with the highest total number of publications **(B)** Number of active journals published per year. **(C)** The dual-map overlay of articles citing on MXene applications research.

Figure 4A shows an overview of active journals in this filed, among which *ACS Applied Materials and Interfaces* was the journal with the highest publication number, scoring a total of 67 papers, followed by *ACS Nano* (*n* = 53) and *Chemical Engineering Journal* (*n* = 47). Figure 4B shows that *ACS Applied Materials and Interfaces* has received extensive attention in the past 2 years. Bradford’s law analysis results also confirmed that *ACS Applied Materials and Interfaces* is a core journal in this research field (Supplementary Figure S3). Supplementary Figure S4 shows that writers from China are more likely to publish on *ACS Applied Materials and Interfaces*, and writers from the United States are more likely to publish in *ACS Nano*.

To obtain a clearer picture of the relationship between different research fields involved in MXene applications, a dual-map overlay of journals was performed to represent the distribution of citing and cited journals (Figure 4C). The colored path represents the relationship between the citing journal (on the left side of the dual-map) and the cited journal (on the right side of the dual-map) (Ke et al., 2020). One major purple path was identified by dual-map overlay. The purple path indicated that studies published in Physics/Materials/Chemistry journals were usually cited in the studies belonging to Chemistry/Materials/Physics.

### 3.5 Co-cited references analysis

Original articles with the most citations are shown in Table 2. The results showed that eight references have been co-cited more than 100 times. The most cited article was “2D metal carbides and nitrides (MXenes) for energy storage” by Anasori et al. (2017) in *Nature Reviews Materials*. Accordingly, this publication was the most cited (*n* = 242) review article on the synthesis, structure and properties of MXene, and their energy storage applications. The next most cited article was Mohamed Alhabeb’s article in *Chemistry of Materials*, “Guidelines for Synthesis and Processing of Two-Dimensional Titanium Carbide (Ti_3_C_2_Tx MXene),” with 223 citations (Lin et al., 2017b), followed by Han Lin’s article in *Nano Letters* “Two-dimensional Ultrathin MXene Ceramic Nanosheets for Photothermal Conversion” (*n* = 187) (Lin et al., 2017a) and Han Lin’s “A Two-Dimensional Biodegradable Niobium Carbide (MXene) for Photothermal Tumor Eradication in NIR-I and NIR-II Bio-Windows” (*n* = 152)" in the *Journal of the American Chemical Society* (Lin et al., 2017a). Among the top 10 most cited articles, there were two reviews and eight studies, of which 50% (4 out of 8) were on the application of MXene to photothermal therapeutics, proving that the photothermal effect in MXene is widely recognized in medical applications.

**TABLE 2:** Top 10 co-cited references and corresponding journals, IF and citations.

Rank	Title of co-cited reference	First author	Journal	IF (2022)	Year	Citations	Type
1	2D metal carbides and nitrides (MXenes) for energy storage	Anasori B	Nature Reviews Materials	76.679	2017	242	Review
2	Guidelines for Synthesis and Processing of Two-Dimensional Titanium Carbide (Ti_3_C_2_TX MXene)	Alhabeb M	Chemistry of Materials	10.508	2017	223	Article
3	Two-Dimensional Ultrathin MXene Ceramic Nanosheets for Photothermal Conversion	Lin H	Nano Letters	12.262	2017	187	Article
4	A Two-Dimensional Biodegradable Niobium Carbide (MXene) for Photothermal Tumor Eradication in NIR-I and NIR-II Bio-windows	Lin H	Journal of the American Chemical Society	16.383	2017	152	Article
5	Two-dimensional transition metal carbides and nitrides (MXenes) for biomedical applications	Huang K	Chemical Society Reviews	60.615	2018	122	Review
6	Electromagnetic interference shielding with 2D transition metal carbides (MXenes)	Shahzad F	SCIENCE	63.714	2017	121	Article
7	Antibacterial Activity of Ti_3_C_2_Tx MXene	Rasool K	ACS Nano	18.027	2016	115	Article
8	Surface Modified Ti_3_C_2_ MXene Nanosheets for Tumor Targeting Photothermal/Photodynamic/Chemo Synergistic Therapy	LiuGY	ACS Applied Materials and Interfaces	10.383	2017	105	Article
9	MXene Ti_3_C_2_: An Effective 2D Light-to-Heat Conversion Material	Li RY	ACS Nano	18.027	2017	98	Article
10	Photoluminescent Ti_3_C_2_ MXene Quantum Dots for Multicolor Cellular Imaging	Xue Q	Advanced Materials	32.086	2017	96	Article

**FIGURE 5:**
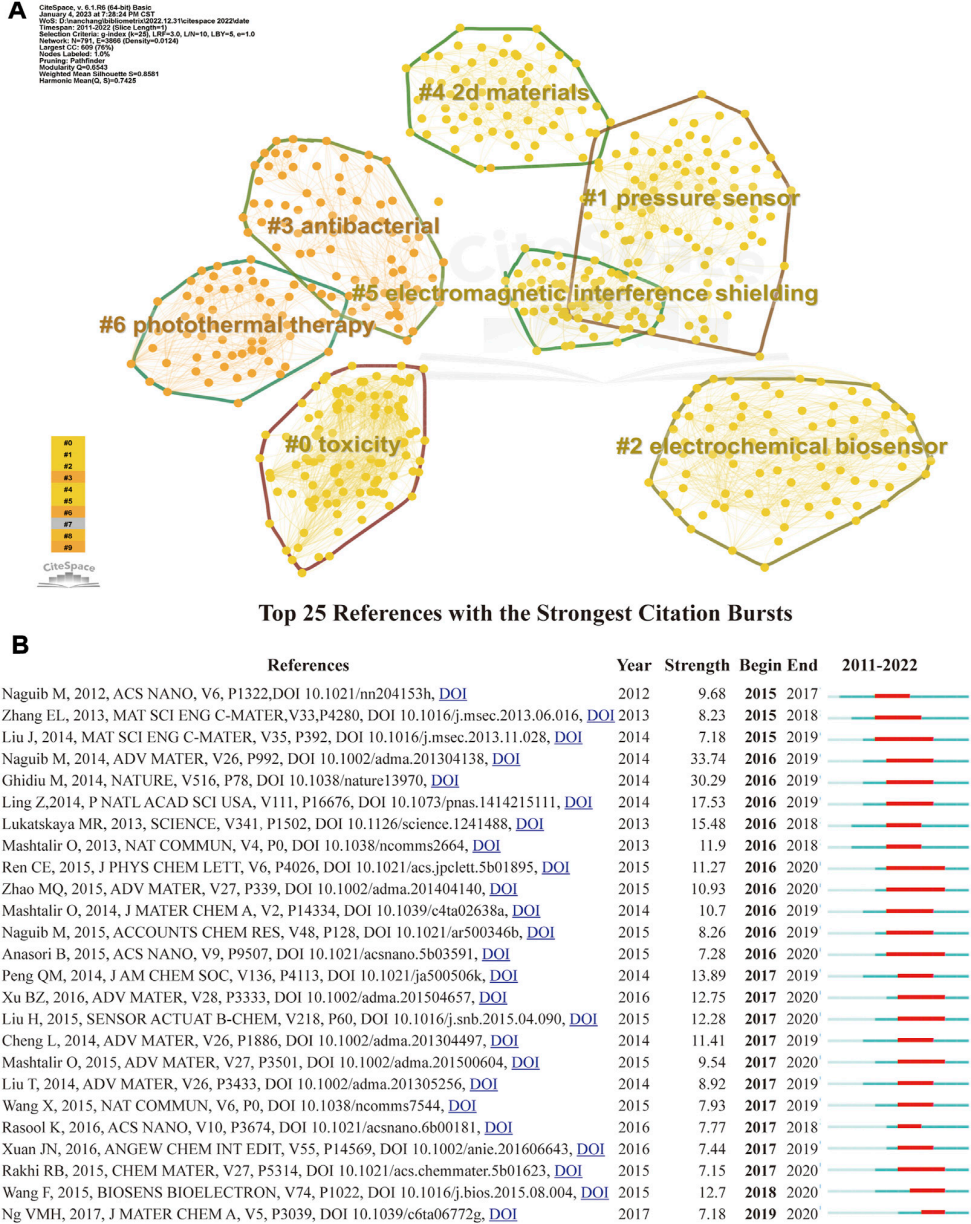
Visualization of co-cited reference analysis. **(A)** Cluster Analysis. **(B)** Representative burst references among top 25 references with the strongest citation bursts.

Cluster analysis allows to classify references and keywords (Ma et al., 2022) and to determine in which areas MXene medical applications are mainly condensed. Modularity Q and weighted mean silhouette S are two vital analysis metrics in the cluster analysis. Values in S > 0.3 indicate that the clustering structure is sufficiently significant and in S > 0.5 indicate convincing agglomeration results. CiteSpace clustering analysis showed a Q value of 0.7425 and an S value of 0.8581, indicating a very significant clustering structure and convincing clustering results. The clustering terms focused on these papers mainly included “toxicity” “pressure sensor” “electrochemical biosensor ""antibacterial”, “2 days materials”, “electromagnetic interference shielding” and “photothermal therapy” (Figure 5A).

“References with the strongest citation bursts” reflect the frequency rate at which particular studies are cited (Wu et al., 2021), representing literature that has been closely followed by scholars within the relevant field over a certain amount of your time (Ke et al., 2020). Figure 5B shows the results of “citation burstiness” analyzed through CiteSpace. Naguib M,2014. ADV MATER,V26, P992 (Naguib et al., 2014) has the highest burst intensity (strength = 33.74, citation burstiness from 2010 to 2014), with the title “25th anniversary article: MXenes: a new family of two-dimensional materials”. Overall, the burstiness strength of the top 25 references ranged from 7.15 to 33.74, while endurance strength was 3–5 years (Figure 5B).

### 3.6 Keyword analysis

**FIGURE 6:**
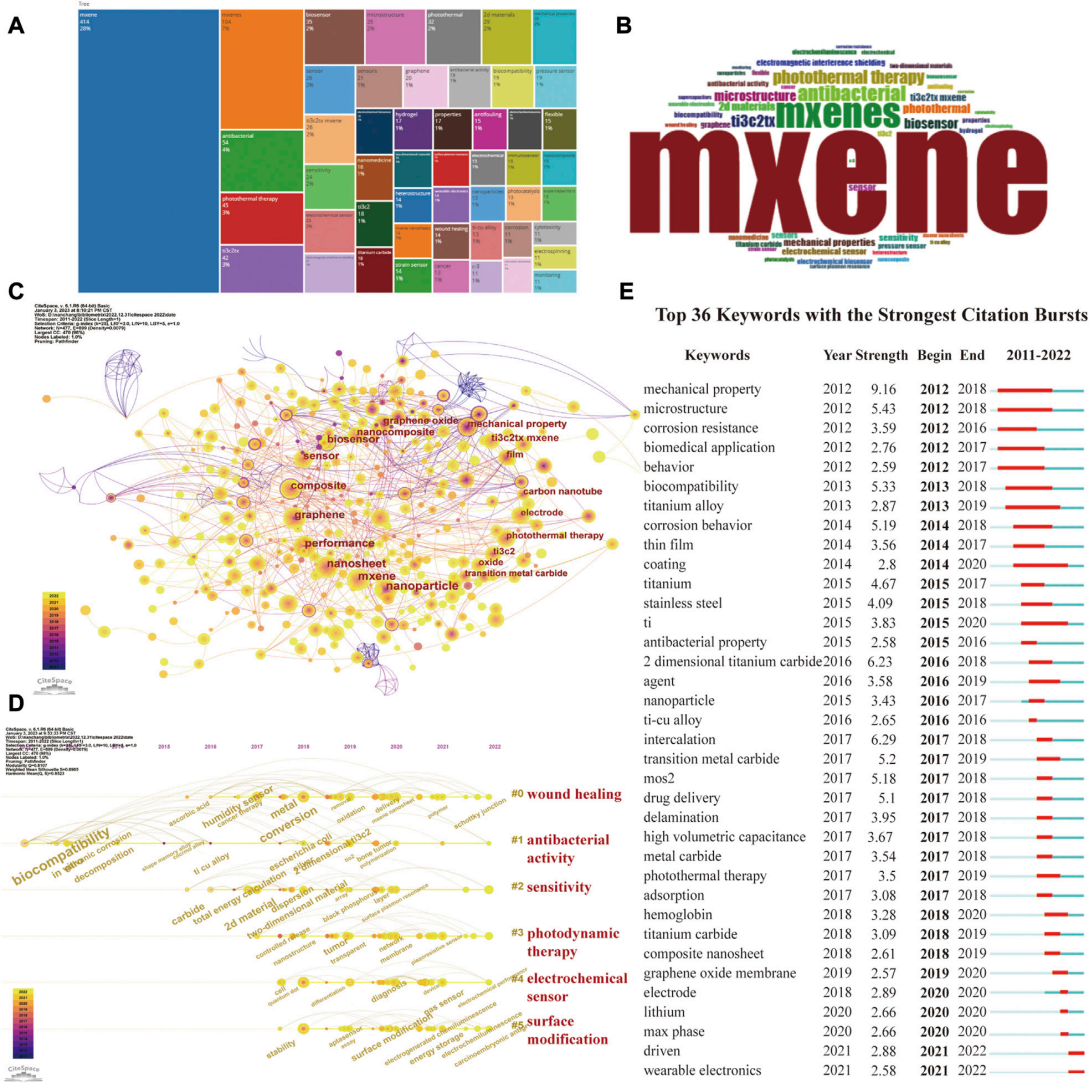
Visual image of keyword analysis. **(A)** The frequency of active keywords. **(B)** World cloud of key word. **(C)** Keywords of MXene for biological application and their interaction. **(D)** Timeline distribution of cluster analysis of keyword. **(E)** Representative burst keywords among top 28 references with the strongest citation bursts.

Keyword often capture the main points of an article, therefore, analyzing key concepts can help researchers realize current research hotspots and trends. The most widely used keywords in MXene medical application are ranked in Figure 6A, with the top keywords identified as MXene, occurring 414 times. We used the size of keywords to show how often they appeared in order to better visualize findings (Figure 6B). In order to understand the progress of MXene applications in biomedical fields, we performed keyword co-occurrence analysis using CiteSpace. The main research hotspots in the corresponding fields were revealed by the keyword co-occurrence network. Thus, we could easily understand the specific research directions in a particular field of research by analyzing keyword co-occurrence. The keyword co-occurrence analysis performed herein showed that antibacterial activity was the keyword with the highest intermediary centrality, demonstrating that MXene antimicrobial applications play an important role in the advancement and development of research in this discipline (Figure 6C, Supplementary Table S2).

The keyword contributions of closely linked keywords can be clustered together according to their similarity to form keyword clusters. The results of keyword clustering analysis by CiteSpace showed a Q value of 0.8523 and an S value of 0.8985, and the main keyword clusters were #0 wound healing, #1 antibacterial activity, #2 sensitivity, #3 photothermal therapy, #4 electrochemical sensor, and #5 surface modification (Supplementary Figure S5). Figure 6D shows the evolution of the keywords over time in the different clusters, showing that the focus of antibacterial activity research evolved from “biocompatibility” to “escherichia coli”.

Emerging academic trends and new topics were shown by keyword bursts. They predicted cutting-edge research directions and revealed potential hotspots in a given field of research. The results presented in Figure 6E showed that the current research frontiers, from 2021 to the present, are “wearable electronics” and “driven,” which further provides researchers with relevant research directions.

## 4 Discussion

**FIGURE 7:**
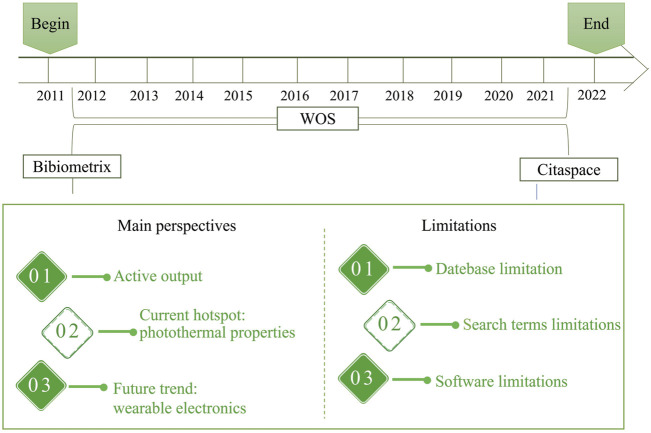
Main perspectives and limitations of the paper.

This study quantitatively analyzed a total of 1,489 publications on medical applications of MXene present in the WoS database collection from a period comprised between 2011 and 2022. The main knowledge areas and emerging trends in medical applications of MXene were analyzed using the visualization tools CiteSpace and Bibliometrix (Figure 7).

The annual output reflects the increasing interest of worldwide researchers in this field of research (Zheng et al., 2022). It shows a yearly increasing trend between 2012 and 2022, reaching a staggering peak in 2022 (number of articles = 629), which indicates a continuous increase in the interest of researchers in this research area. A total of 63 countries contributed so far to this research area. The top three countries were China, the United States and India. Interestingly, China, as the developing country with the highest number of publications, was found to have less interaction with other countries and revealed a lower intermediary centrality. This result is consistent with the research results of Saravanan et al., 2022, in 2022, that is, although China has the highest publication rate, the article citation rate is still slightly lower than that of the United States. Academic cooperation can promote the output of research results (Wang et al., 2022), which suggests that China needs to expand cooperation and strengthen synergy with other countries. Sichuan University is the institution that publishes the most papers. Indeed, the institute has been committed to the research of MXene medical application for a long time. The latest research paper is the combination of MXene and polyvinylidene fluoride (MXene/PVDF) using electrospinning technology (Fu et al., 2023). Briefly, MXene/PVDF shows good biocompatibility, promotes the differentiation of osteoblasts, and provides an innovative and very suitable strategy for bone regeneration.

This study found that among the top 10 core journals, *ACS Applied Materials* and *Interfaces* (*n* = 67), *ACS Nano* (*n* = 53) and *Chemical Engineering Journal* (*n* = 47) had the highest number of published articles. It shows that *ACS Applied Materials* and *Interfaces* is an influencial journal in the field of MXene biological application and has great impact in this field. In this journal, Ti_3_C_2_-DOX nanomaterials reported by Liu et al. (2017) can achieve efficient killing of tumor cells and tissues through photothermal/photodynamic/chemical synergy (Liu et al., 2017). This article has a high citation rate and is a basic research on the medical application of MXene, which has been recognized by the majority of researchers (Table 2).

In addition, this study found that nearly half (*n* = 4) of the top 10 most cited articles on MXene medical applications focus on the application of MXene PPT. PTT also appeared in the co-cited reference cluster analysis and keyword cluster analysis, indicating that PTT is the focus of current research. PTT entails the use of photothermal transducers (PTA), which can be used as therapeutic tools by converting light energy into heat (Liu et al., 2019). As a non-invasive treatment method, PTT has the advantages of low adverse effects, high specificity and reproducibility, and is widely used due to its antimicrobial and antitumoral capacities (Chen et al., 2020; Xu et al., 2021). There are many PTA materials, such as carbon-based nanomaterials, noble metal nanoparticles, conjugated polymers, and metal-organic frameworks. Among these, MXene, a 2D nanosheet material, stands out as one of the most widely studied and promising candidates due to its high aspect ratio, atomic thickness, excellent photothermal properties, low toxicity and ultra-high dispersion in aqueous systems (Hao et al., 2022; Szuplewska et al., 2019). For example, He et al. (2022) loaded adriamycin (doxorubicin, DOX) on Ti_3_C_2_Tx and then combined it with DNA hydrogels to establish a platform for efficient photothermal-chemical synergistic cancer therapy.

In addition to the widely used PTT applications of MXene, some emerging research areas are becoming topics of interest for researchers. Our keyword analysis indicated that some edgy applications are at the forefront of current research, namely, “wearable electronics,” a class of physical sensors that can sense external physical and chemical signals and convert them into electrical signals (Jia et al., 2022). Compared with other materials, MXenes and their composites exhibit good mechanical flexibility and tensile properties, making them widely used in wearable sensors, energy storage and electromagnetic shielding (Xin et al., 2020). For example, Li et al. (2022a) fabricated a Ti_3_C_2_Tx MXene-based wireless facemask to achieve real-time dynamic detection of Breath acetone (BrAC) through a filter-detect-calibrate-transfer system with a hierarchical design.

Despite the novelty of the bibliometric analysis provided herein, our study still has some limitations: 1) although WoS is the world’s largest and most comprehensive database of information resource collections (Li and Song, 2022), it does not contain all research literature in this field; 2) due to the wide variety of MXene nanomaterials, experimental methods can yield about twenty different compositions of MXenes (Huang et al., 2020), and this was not accounted in this study. Although we expanded the search for MXene nanomaterials as much as possible, some studies on the medical applications of MXene nanomaterials were not included; 3) The bibliometric software used for analysis also presents some limitations. The software is only able to analyze information such as keywords, references, authors, and institutions, and lacks the analysis of specific content present within the included research articles.

## 5 Conclusion

This study provides the first attempt to visualize and analyze MXene medical application research articles based on CiteSpace and Biblimetrix. Altogether, our results show that the number of publications on medical applications of MXene nanomaterials is rising, which indicates that this field of research is being widely valued nowadays. Moreover, the institutions and authors with the highest number of publications are mainly from China. The number of publications in this field is also much higher in China compared to that of other countries, indicating that China has contributed the most in this research field. However, the intermediary centrality of China appeared low, suggesting that China may need to strengthen international cooperation. Interestingly, cluster and reference analysis revealed that current research hotspots in this field are mainly focused on MXene-mediated PTT methods for cancer and bacteria whereas future research hotspots will focus on wearable devices. Overall, this study guides the objective visualization and analysis of the medical applications of MXene and provides a bibliometric reference for related personnel.

## Data Availability

The original contributions presented in the study are included in the article/Supplementary Material, further inquiries can be directed to the corresponding author.
