# Corrigendum: MXene nanomaterials in biomedicine: a bibliometric perspective

**DOI:** 10.3389/fbioe.2023.1260854

**Published:** 2023-09-04

**Authors:** Runying Guo, Daorun Hu, Danrui Liu, Qingkun Jiang, Jiaxuan Qiu

**Affiliations:** ^1^ Department of Stomatology, First Affiliated Hospital of Nanchang University, Nanchang, China; ^2^ Medical College, Nanchang University, Nanchang, China

**Keywords:** MXene, biomaterials, biological application, bibliometric review, visualization

In the published article, there was an error in Figure 2C as published. In the Figure 2C section of the article, we encountered some errors in color rendering when visualizing the cooperation relationship between countries. These issues may lead to the readers’ incorrect understanding of the results. The corrected Figure 2 and its caption Figure 2 appear below.

**FIGURE 2:**
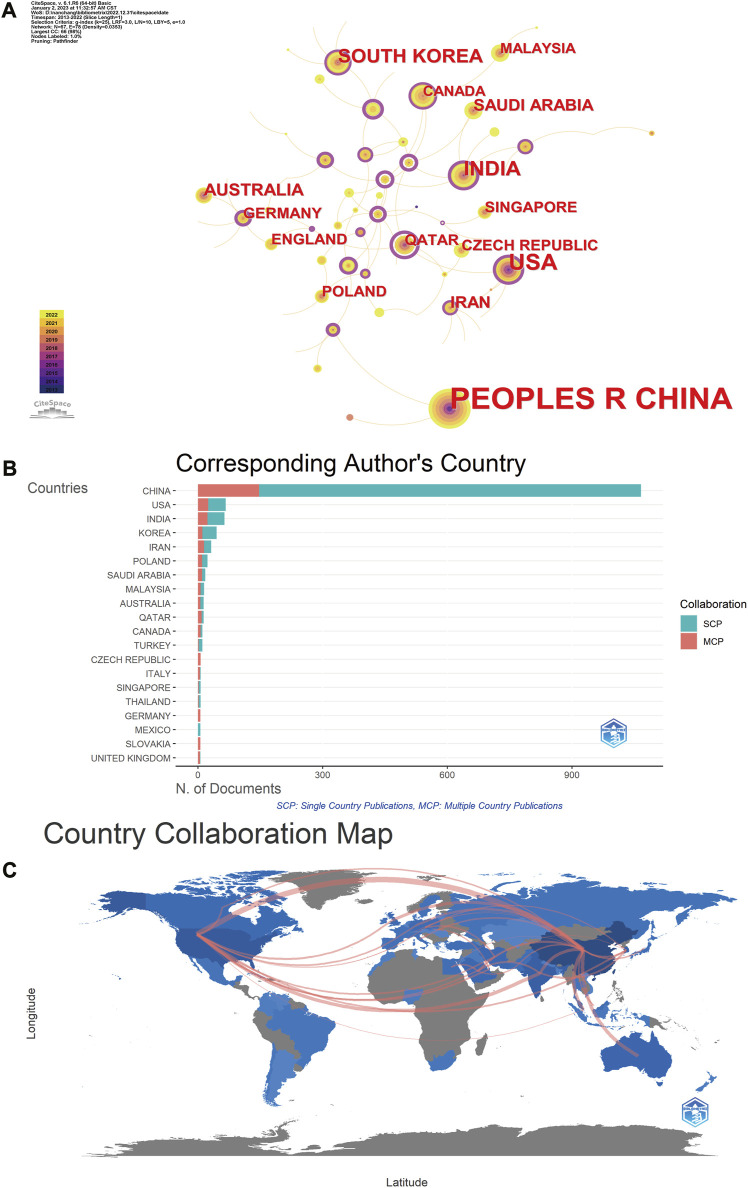
Visualization of the network map of countries. **(A)** Country distribution of MXene medical application. **(B)** Cooperation in the country where the corresponding author is located. **(C)** Map of cooperation between countries. SCP, single country publications; MCP, multiple country publications.

The authors apologize for this error and state that this does not change the scientific conclusions of the article in any way. The original article has been updated.

