# Supplementary material for: MXene nanomaterials in biomedicine: A bibliometric perspective
Source: Front Bioeng Biotechnol. 2023 Apr 19;11:1184275. doi: 10.3389/fbioe.2023.1184275 (PMC10154466; doi:10.3389/fbioe.2023.1184275)
Supplement: Supplementary file 1 [file DataSheet1.doc]

**MXene nanomaterials in biomedicine: a bibliometric perspective**

**Runying Guo ^1,2^, Daorun Hu ^1,2^, Danrui Liu ^1,2^, Qingkun Jiang ^1^, Jiaxuan Qiu ^1,*^**

^1^Department of Stomatology, The First Affiliated Hospital of Nanchang University, Nanchang, China

^2^Medical College, Nanchang University, Nanchang, China

*** Correspondence:** Jiaxuan Qiu: qiujiaxuan@163.com (J. Qiu)

# Supplementary data


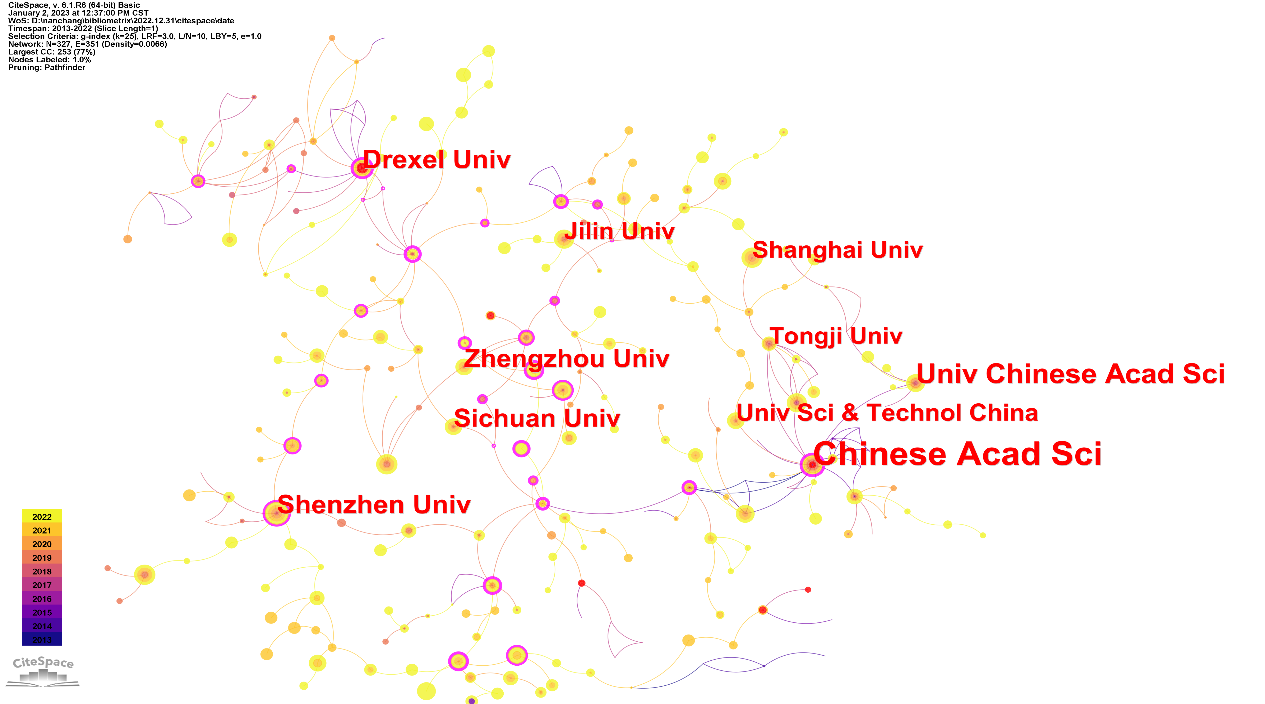


Supplementary Figure 1. Visual analysis of institutional collaboration network.


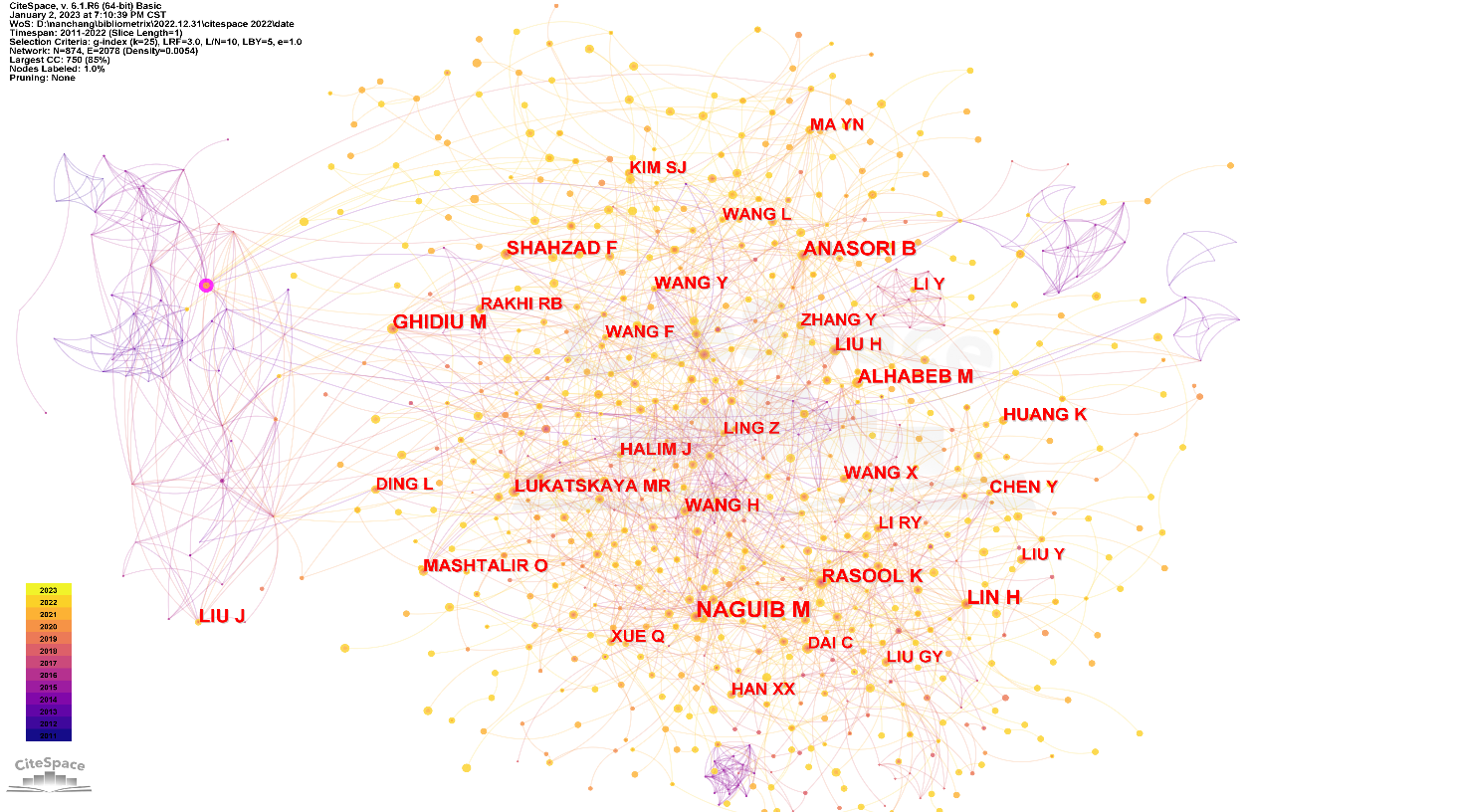


Supplementary Figure 2. Visual analysis of co-cited authors.


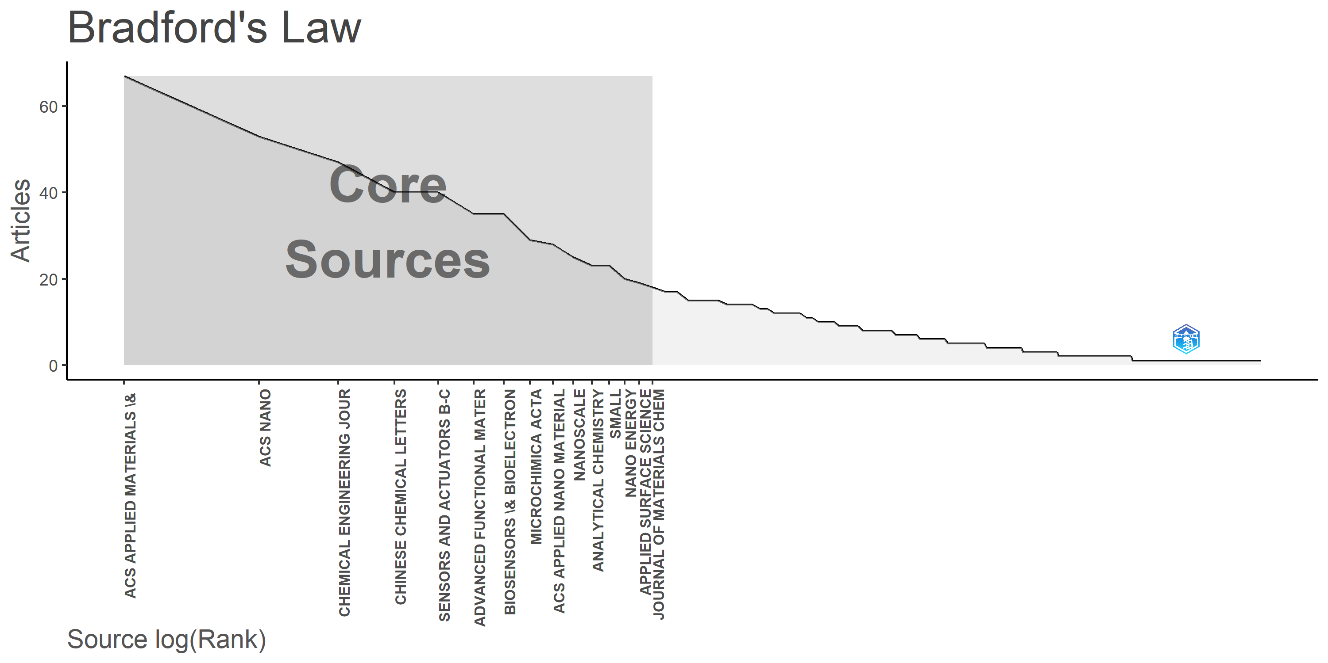


Supplementary Figure 3. Law of document dispersion
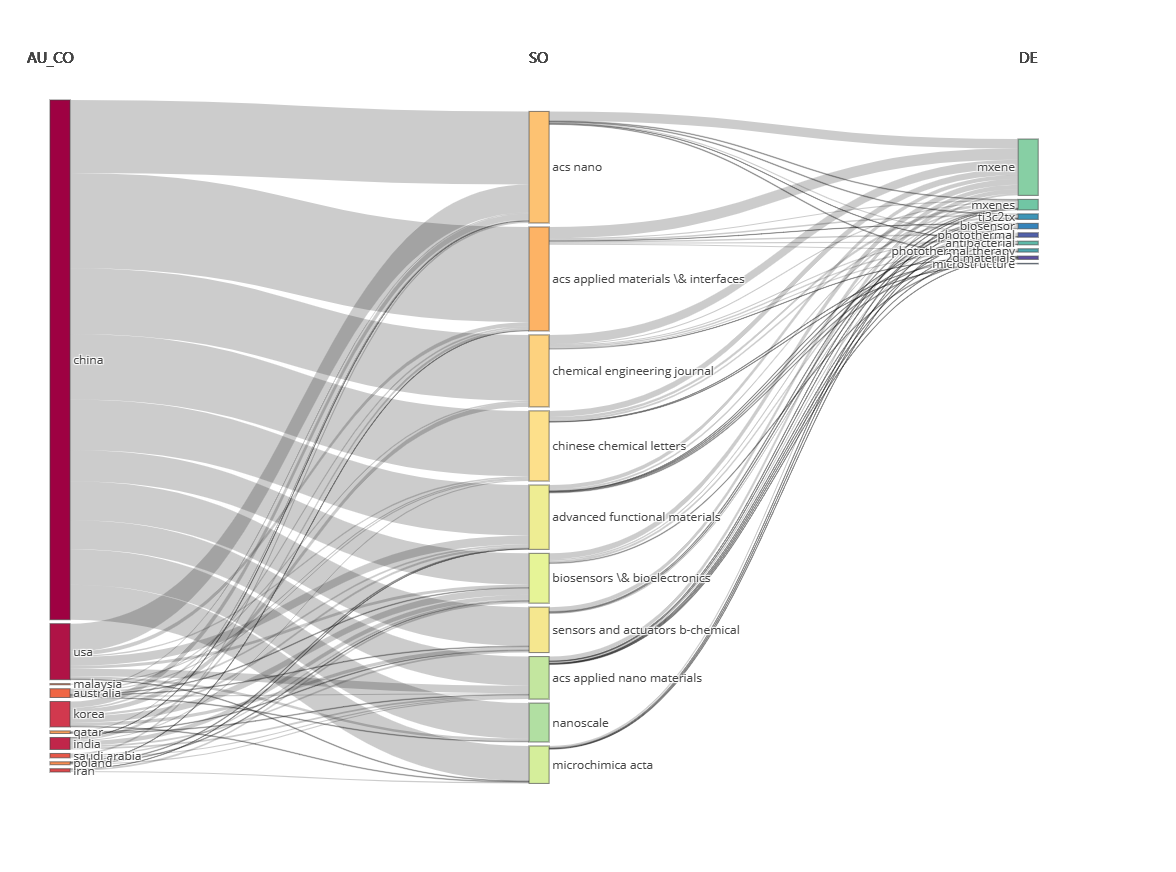
Supplementary Figure 4. Association diagram of countries, journals and keywords.


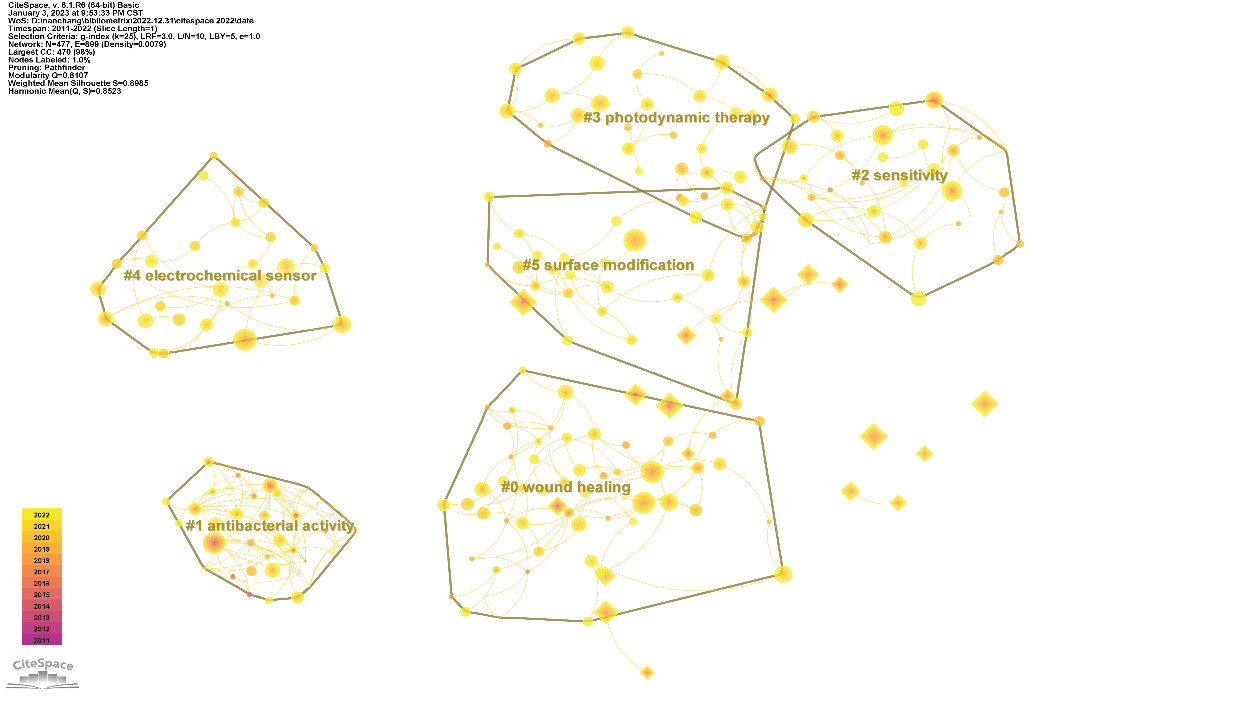


Supplementary Figure 5. Keywords clustering result.

**Supplementary Table 1 Annual-Production.**

| **Year** | **Articles Number** | **Percent (%)** |
| --- | --- | --- |
| 2011 | 2 | 0.134 |
| 2012 | 4 | 0.269 |
| 2013 | 5 | 0.336 |
| 2014 | 12 | 0.806 |
| 2015 | 12 | 0.806 |
| 2016 | 17 | 1.142 |
| 2017 | 29 | 1.948 |
| 2018 | 60 | 4.030 |
| 2019 | 120 | 8.059 |
| 2020 | 220 | 14.775 |
| 2021 | 379 | 25.453 |
| 2022 | 629 | 42.243 |

**Supplementary Table 2.** Top 10 keyword with the highest centrality score.

| Rank | Centrality | Keyword |
| --- | --- | --- |
| 1 | 0.31 | antibacterial activity |
| 2 | 0.3 | corrosion resistance |
| 3 | 0.26 | anode |
| 4 | 0.22 | conversion |
| 5 | 0.2 | biocompatibility |
| 6 | 0.2 | metal |
| 7 | 0.19 | 2 dimensional titanium carbide |
| 8 | 0.18 | agent |
| 9 | 0.17 | biosensor |
| 10 | 0.15 | fabrication |
